# Teacher stress, anxiety and depression at the beginning of the academic year during the COVID-19 pandemic

**DOI:** 10.1017/gmh.2021.14

**Published:** 2021-04-12

**Authors:** María Dosil Santamaría, Nahia Idoiaga Mondragon, Naiara Berasategi Santxo, Naiara Ozamiz-Etxebarria

**Affiliations:** 1 Department of Research and Diagnostic Methods in Education, University of the Basque Country UPV/EHU, Leioa, Spain; 2 Department of Developmental and Educational Psychology, University of the Basque Country UPV/EHU, Leioa, Spain; 3 Department of Didactics and School Organization, University of the Basque Country UPV/EHU, Leioa, Spain

**Keywords:** Age, anxiety, chronic disease, COVID-19, depression, education, gender, stress

## Abstract

**Background:**

Faced with the situation of COVID-19, teachers are dealing with new measures, insecurity and a lack of clear guidelines. The aim of this study is to analyse the levels of stress, anxiety and depression of teachers in the north of Spain.

**Methods:**

This study was conducted with 1633 teachers from the Department of Education of the Basque Autonomous Community (BAC) and Navarre, all of whom are professionals working in various educational centres, from nursery education to university studies, with an average age of 42 years (*M* = 42.02; s.d. = 10.40). The Spanish version of the Depression Anxiety and Stress Scale-21 was used.

**Results:**

The results show that a high percentage of teachers have symptoms of stress, anxiety and depression. Women show significantly more symptoms of stress and anxiety than men, those who have children have more depressive symptoms than those who do not, and people with chronic pathology or those who live with others with chronic pathology have more stress, anxiety and depression.

**Conclusions:**

This study indicates the importance of attending to the mental health of teachers, particularly women, those who have children, and those who have a chronic pathology or a family member with a chronic pathology.

## Introduction

In December 2019, the first outbreak of COVID-19 occurred in Wuhan (Hubei, China). From that moment, the virus began to spread worldwide in an imperious and uncontrolled way. In response to this unprecedented pandemic, the World Health Organization (WHO) declared the COVID-19 outbreak a public health emergency of international concern on 30 January 2020 (Ho *et al*., 2020).

Spain has been – and continues to be – one of the European countries most affected by this pandemic. COVID-19 began to spread in the country at the end of February 2020, and, as a consequence, on 14 March the Spanish government declared a state of emergency (Government of Spain, 2020). During that state of emergency until the end of April, citizens (including children) were completely forbidden to leave their homes except to cover basic needs (Lucas, 2020*a*). As a consequence, all teaching activities (from preschool to university education) were transformed to an online modality.

This online or telematic teaching was maintained during the 2019–2020 school year (the school year in Spain starts in September and finishes in June/July), and therefore the majority of students and teachers did not return to school since their abrupt departure on 13 March. However, it is the case at the end of June an exception was made for students in their final years of high school, who were allowed to return to the classroom to prepare for their college entrance exams (Torres, 2020).

Studies conducted during those months of lockdown have pointed out the importance of reopening classrooms as soon as the health situation allows (Francis and Pegg, 2020; UNESCO, 2020*a*). In fact, several studies have stated that not being able to attend school was having a negative impact on the emotional, physical, social and academic well-being of children (Armitage and Nellums, 2020; Berasategi *et al*., 2020; Burgess and Sievertsen, 2020; Idoiaga *et al*., 2021; Imran *et al*., 2020; Kleinberg *et al*., 2020).

Moreover, teachers have not been immune to the effects of the pandemic. In fact, UNESCO (2020*a*) has already identified confusion and stress among teachers as one of the adverse consequences of school closures due to the abruptness of such closures, the uncertainty about their duration and relatively poor knowledge of distance education.

Indeed, several studies have shown that even at the beginning of the pandemic, teachers accumulated high levels of stress accompanied by symptoms of anxiety, depression and sleep disturbance, particularly as a result of having to teach online (Ng, 2007; Al Lily, 2020; Besser *et al*., 2020). Some studies have found that the use of Information and Communication Technologies (ICT) for working at home can create feelings of tension, anxiety, exhaustion and decreased job satisfaction (Cuervo *et al*., 2018), and in times of a pandemic these were the only tools available to teachers. However, numerous studies conducted during the COVID-19 crisis have suggested that other variables such as age (González-Sanguino *et al*., 2020; Nwachukwu *et al*., 2020; Picaza *et al*., 2020), gender (Lai *et al*., 2020; Talevi *et al*., 2020; Ozamiz-Etxebarria *et al*., 2020*a*), having children (Cameron *et al*., 2020; Fitzpatrick *et al*., 2020) or previously suffering from chronic illnesses (Dosil Santamaría *et al*., 2020) also affect the population's levels of stress, depression and anxiety. In particular, the results of most of these studies appear to indicate that prior to the onset of COVID-19 those who had more psychological symptoms were young people, women, people with chronic diseases and people with children.

It has been found that young adults have been particularly vulnerable to high levels of psychological symptoms in response to the COVID-19 outbreak (Gao *et al*., 2020; Huang and Zhao, 2020; Lai *et al*., 2020; Qiu *et al*., 2020). These high levels of distress among young people could be due to the fact that they tend to gather a large amount of information from social media, which could easily trigger stress (Achdut and Refaeli, 2020).

Furthermore, and as previously noted, gender could be another variable to be considered in the psychological response to the pandemic. In fact, many studies have shown that women appear to present more severe symptoms of depression, anxiety and distress in comparison with men (Gao *et al*., 2020; Huang and Zhao, 2020; Lai *et al*., 2020; Liu *et al*., 2020; Qiu *et al*., 2020). It is also worth noting that the teaching profession, especially at the earliest stages, is a particularly hyper-feminised field (Corral, 2016). Similarly, the profile of female teachers is intrinsically linked to care (Vendrell *et al*., 2008). This linkage may have consequences for the emotional state of female teachers, as the tendency to identify care with the non-professional sphere may discredit their work (Wharton, 2005).

In addition, people with children and those without emotional support could be particularly prone to showing adverse psychological symptoms, according to several studies (Cameron *et al*., 2020; van der Velden *et al*., 2020).

Finally, recent studies have also shown that these increased levels of stress, anxiety and depression resulting from COVID-19 could be particularly prevalent among people with a history of health problems (Applegate and Ouslander, 2020; Dong *et al*., 2020; Ozamiz *et al*., 2020*b*).

Amid this context of uncertainty, the 2020–2021 school year has approached without any clear decision on how it will be played out (Zafra, 2020*a*). In fact, since mid-August, with the imminent arrival of the new school year 2020–2021, the teaching community, society and politicians have been engaged in a public debate on how to address the situation, without reaching consensus, while there has been a lack of clear government guidelines (Marcos, 2020). On 27 August, the Spanish Government and the Autonomous Communities at the Education Sector Conference agreed on the main measures that should be implemented for the return to school (Sanchez, 2020). But, the issue of how to apply these measures with the available resources was clearly a challenge and a source of concern just 1 week before the beginning of the school year on 7th September (Lucas, 2020*b*). In September, classes returned, but the teachers and school centres were not satisfied about the guidelines that they were required to follow (Atlas, 2020). Moreover, the responsibility for the health measures was put into the hands of each school, high school and university (Zafra, 2020*b*).

Although this pandemic has led us to conclude that the presence of students in schools is necessary for adequate learning and human relations (Caffo *et al*., 2020), the conditions that have been faced by teachers upon the start of the new school year 2020–2021 have not been entirely satisfactory. In fact, in the face of the lack of coordination, leadership and resources, several schools and teachers expressed their uneasiness both in the press and in the streets, even calling for a teachers' strike on 15th September in the Autonomous Community of the Basque Country (Navarro, 2020).

Within the scientific community, it has been pointed out that the immediate priority of the research community at this unprecedented time should be to reduce mental health problems and support the wellbeing of vulnerable groups. Indeed, the COVID-19 pandemic may have a long-lasting impact on teachers and teaching activities, and, as a result, on children and adolescents (Holmes *et al*., 2020). Therefore, by considering how teachers are coping with the return to school in times of a pandemic we might be in a better position to put in place the relevant support structures that may be needed (Holmes *et al*., 2020; Dalton *et al.*, 2020; Wang *et al*., 2020).

Therefore, the main objective of this study was to analyse how teachers have coped with the return to school in times of a pandemic and to what extent they suffer from stress, anxiety and depression disorders. Although it is expected that teachers will particularly suffer from stress symptomatology, we also anticipate that the levels of anxiety, depression and stress will not be homogeneous in the sample. That is, it is predicted that there will be specific and significant differences between groups. In particular, we aim to explore possible differences according to gender, age, having children, having a chronic disease or living with people with chronic diseases. It is anticipated that women may have higher levels of adverse psychological symptoms than men. In addition, younger teachers and those with a previous history of chronic illness and with family members with chronic diseases are also expected to show higher levels of stress, anxiety and depression.

## Method

### Participants

This study was conducted with a total sample of 1633 teachers from the Department of Education of the Basque Autonomous Community (BAC) and Navarre, all of whom are professionals working in different educational centres. Of the sample, 18.9% (*n* = 309) were professionals working in early childhood education, 32.5% (*n* = 530) in primary education, 30.1% (*n* = 491) in secondary education, 5.5% (*n* = 89) in high school, 5.6% (*n* = 91) and 7.5% (*n* = 123) in university studies. The minimum age was 23 years and the maximum was 67 years. Of these people, 1293 were women (*M* age = 42.6; s.d. = 9.96) and 330 were men (*M* age = 42.02; s.d. = 10.40). Of the total participants, 47.3% (*n* = 772) had school-age children, and 16.7% (*n* = 273) had a chronic disease. In addition, 18.1% (*n* = 296) of the sample indicated living with someone with a chronic illness. In relation to having had a family member with COVID-19, 35.2% (*n* = 574) said yes.

### Instruments

A questionnaire was designed to gather sociodemographic (gender and age) and socio-personal data such as whether they had school-age children, whether they suffered from chronic disease, or whether they had been in contact with people infected with the COVID-19 virus.

The Spanish version of the Depression Anxiety and Stress Scale-21 (DASS-21; Ruiz *et al*., 2017) was employed. This instrument consists of 21 items with four response options (from 0 = did not occur to 3 = occurred a lot or most of the time) that are grouped into three factors: depression, anxiety and stress: depression (items: 3, 5, 10, 13, 16, 17 and 21), anxiety (items: 2, 4, 7, 9, 15, 19 and 20) and stress (items: 1, 6, 8, 11, 12, 14 and 18). The following cut-off points were used: no symptoms, mild, moderate, severe and extremely severe symptoms. Regarding the reliability of the scale, the Cronbach's alpha coefficient was *α* = 0.76 for the depression scale, *α* = 0.82 for the anxiety scale and *α* = 0.75 for the stress scale.

### Procedure

The study was approved by the Ethics Committee of the UPV/EHU (code M10/2020/070). The participants were recruited through non-probabilistic snowball sampling. Responses were collected via an online questionnaire between 5 and 28 September 2020 (beginning of the school year) with a prior request for consent for subject participation. The questionnaire explained both the objectives of the study and the procedure to be followed. In addition, all the requirements established by the Organic Law 15/99 on the Protection of Personal Data were followed for the collection of data.

### Data analysis

The data were analysed using the statistical program IBM SPSS Statistics for Windows, Version 26.0 (Armonk, NY, USA). Two assumptions of normality and homogeneity of variations were then confirmed before the corresponding analysis to decide whether parametric or non-parametric tests were used. Specifically, the critical level of *p* < 0.05 of the Kolmogorov–Smirnov statistic was analysed to determine the distribution of the data for the analysis of group differences.

It should be noted that the categories of depression, anxiety and stress were categorised using the cut-off scores of the instrument in order to obtain the various levels (mild, moderate, severe and extremely severe). First, both the frequencies and the percentages of the socio-demographic variables were described. In the case of gender, only the feminine and masculine gender were used, since ‘other gender’ was represented by only 10 participants. For the remaining variables, the total number of participants was used.

Subsequently, a multivariate analysis of variance (MANOVA) was performed, treating the dependent variables as continuous (levels of depression, anxiety and stress) according to the sociodemographic variables analysed.

## Results

Table 1 shows the frequency and percentages of the variables gender and age according to the category of symptoms. The higher frequencies and percentages in women are in the moderate category. In the case of men, the same pattern was observed except in the case of stress, where more participants reported mild stress (3.5%, *n* = 56). In the case of age and depression, the highest percentage of symptoms was found in the moderate category, except for the oldest (<36 years), who showed the highest percentage in the mild category. However, in the case of anxiety, this was found to be moderate across all age ranges. With regards to stress, the patterns vary depending on age, with the youngest and oldest participants showing moderate stress.

**Table 1:** Frequencies and percentages of teachers in different symptomatology's (mild, moderate, severe and extremely severe) according to gender and age

		Total (%)	Female	Male	23–30 years	31–35 years	>36 years
Total sample (*N* = 1633)		*n* (%)	*n* (%)	*n* (%)	*n* (%)	*n* (%)	*n* (%)
Depression	Mild	12.7% (*n* = 206)	11% (*n* = 179)	1.7% (*n* = 27)	1.5% (*n* = 24)	2% (*n* = 33)	9.2% (*n* = 151)
(32.2%, *n* = 522)	Moderated	11.9% (*n* = 193)	8.6% (*n* = 140)	3.3% (*n* = 53)	1.6% (*n* = 26)	1.8% (*n* = 29)	8.6% (*n* = 140)
	Severe	4.3% (*n* = 70)	3.2% (*n* = 52)	1.1% (*n* = 18)	0.3% (*n* = 5)	1.7% (*n* = 12)	3.2% (*n* = 53)
	Extremely severe	3.3% (*n* = 53)	2.8% (*n* = 46)	0.4% (*n* = 7)	0.2% (*n* = 4)	0.5% (*n* = 8)	2.5% (*n* = 41)
Anxiety	Mild	12.3% (*n* = 199)	9.5% (*n* = 154)	2.8% (*n* = 45)	1.4% (*n* = 23)	1.8% (*n* = 29)	9.1% (*n* = 148)
(49.3%, *n* = 800)	Moderated	21.3% (*n* = 345)	18.1% (*n* = 294)	3.1% (*n* = 51)	3.1% (*n* = 51)	3.1% (*n* = 51)	15.2% (*n* = 249)
	Severe	7.6% (*n* = 124)	6.4% (*n* = 104)	1.2% (*n* = 20)	0.9% (*n* = 15)	1.2% (*n* = 20)	5.5% (*n* = 89)
	Extremely severe	8.1% (*n* = 132)	7.1% (*n* = 116)	1.0% (*n* = 16)	1% (*n* = 16)	1.6% (*n* = 26)	5.6% (*n* = 91)
Stress	Mild	16% (*n* = 260)	12.6% (*n* = 204)	3.5% (*n* = 56)	1.8% (*n* = 29)	3.5% (*n* = 57)	10.8% (*n* = 177)
(50.4%, *n* = 818)	Moderated	15.8% (*n* = 256)	13.4% (*n* = 218)	2.3% (*n* = 38)	2.3% (*n* = 37)	1.8% (*n* = 29)	11.9% (*n* = 194)
	Severe	14.1% (*n* = 229)	11.8% (*n* = 192)	2.3% (*n* = 37)	1.2% (*n* = 20)	2% (*n* = 33)	10.8% (*n* = 177)
	Extremely severe	4.5% (*n* = 73)	3.6% (*n* = 59)	0.9% (*n* = 14)	0.4% (*n* = 7)	0.7% (*n* = 11)	3.4% (*n* = 56)

Table 2 shows the socio-personal variables and the different categories of symptoms (mild, moderate, severe and extremely severe).

**Table 2:** Frequencies and percentages of teachers in different symptomatology's (mild, moderate, severe and extremely severe) according to socio-personal variables

Total sample (*N* = 1633)	Having children *n* (%)			Chronically ill *n* (%)		Living with a chronically ill person *n* (%)		Family member with COVID-19 *n* (%)	
	Yes		No	Yes	No	Yes	No	Yes	No
Depression	Mild	7.8% (*n* = 127)	5% (*n* = 81)		3% (*n* = 49)	9.7% (*n* = 159)	2.6% (*n* = 43)	10.1% (*n* = 165)	5.3% (*n* = 86)	7.4% (*n* = 121)
(32.2%, *n* = 522)	Moderated	6.6% (*n* = 107)	5.4% (*n* = 88)		2% (*n* = 33)	9.9% (*n* = 162)	2.6% (*n* = 42)	9.4% (*n* = 153)	3.9% (*n* = 64)	8% (*n* = 131)
	Severe	2% (*n* = 33)	2.3% (*n* = 37)		0.9% (*n* = 15)	3.4% (*n* = 55)	1.2% (*n* = 19)	3.1% (*n* = 51)	1.4% (*n* = 23)	2.9% (*n* = 47)
	Extremely severe	1.7% (*n* = 28)	1.5% (*n* = 25)		0.9% (*n* = 15)	2.3% (*n* = 38)	1% (*n* = 16)	2.3% (*n* = 37)	1.3% (*n* = 21)	2% (*n* = 32)
Anxiety	Mild	5.9% (*n* = 97)	6.3% (*n* = 103)		1.5% (*n* = 24)	10.8% (*n* = 176)	2.1% (*n* = 35)	10.1% (*n* = 165)	4.7% (*n* = 77)	7.5% (*n* = 123)
(49.3%, *n* = 800)	Moderated	9.4% (*n* = 153)	12.1% (*n* = 198)		5.5% (*n* = 89)	16% (*n* = 262)	4.2% (*n* = 69)	17.3% (*n* = 282)	7.8% (*n* = 127)	13.7% (*n* = 223)
	Severe	3.6% (*n* = 59)	4% (*n* = 65)		1.1% (*n* = 18)	6.5% (*n* = 106)	1.7% (*n* = 27)	5.9% (*n* = 97)	3.4% (*n* = 56)	4.2% (*n* = 68)
	Extremely severe	3.9% (*n* = 64)	4.2% (*n* = 69)		2.3% (*n* = 38)	5.8% (*n* = 95)	2.3% (*n* = 38)	5.8% (*n* = 95)	3.1% (*n* = 50)	5% (*n* = 82)
Stress	Mild	17.2% (*n* = 117)	8.9% (*n* = 146)		2.8% (*n* = 46)	13.3.% (*n* = 217)	2.7% (*n* = 44)	13.4% (*n* = 219)	6.1% (*n* = 99)	10% (*n* = 163)
(50.4%, *n* = 818)	Moderated	7.2% (*n* = 118)	8.7% (*n* = 142)		2.8% (*n* = 45)	13.2% (*n* = 215)	3.1% (*n* = 51)	12.8% (*n* = 209)	6.1% (*n* = 100)	9.8% (*n* = 160)
	Severe	7.7% (*n* = 126)	6.4% (*n* = 104)		3.2% (*n* = 53)	10.8% (*n* = 177)	3.7% (*n* = 61)	10.3% (*n* = 169)	5.2% (*n* = 85)	8.8% (*n* = 144)
	Extremely severe	2.4% (*n* = 40)	2.1% (*n* = 34)		1.3% (*n* = 21)	3.2% (*n* = 53)	1.4% (*n* = 23)	3.1% (*n* = 51)	1.8% (*n* = 30)	2.7% (*n* = 44)

A MANOVA was conducted to explore whether the variables measured in the *ad hoc* questionnaire were related to levels of depression, anxiety and stress. Table 3 shows the descriptive results and Table 4 shows which of these relationships were statistically significant.

**Table 3:** Means and typical deviations in depression, anxiety and stress according to the collected socio-personal variables

	Depression			Anxiety			Stress		
	*M*	s.d.	*n*	*M*	s.d.	*n*	*M*	s.d.	*n*
Sex									
Female	3.77	4.05	1293	4.29	3.60	1293	8.09	4.58	1293
Male	3.66	4.02	330	3.27	3.31	330	6.96	4.89	330
Age									
23–30	3.24	3.73	206	4.16	3.59	206	7.17	4.55	206
31–35	3.74	4.17	246	4.41	3.93	246	7.81	4.59	246
>36	3.85	4.05	1181	4.02	3.49	1181	8.02	4.70	1181
Having children									
Yes (*n* = 861, 52.7%)	3.83	4.04	861	4.05	3.60	772	8.10	4.81	772
No (*n* = 772, 47.3%)	3.66	4.03	772	4.13	3.55	861	7.67	4.54	861
Chronically ill									
Yes (*n* = 27 316.7%)	4.58	4.46	273	5.23	3.98	273	9.16	4.80	273
No (*n* = 1360, 83.3%)	3.59	3.92	1360	3.87	3.44	1360	7.62	4.61	1360
Living with a chronically ill person									
Yes (*n* = 296, 18.1%)	4.67	4.51	296	4.91	4.08	296	9.09	4.85	296
No (*n* = 1337, 81.9%)	3.55	3.89	1337	3.91	3.43	1337	7.61	4.59	1337
Family member with COVID-19									
Yes (*n* = 574, 35.2%)	3.93	3.98	574	4.37	3.62	574	8.25	4.58	574
No (*n* = 1059, 64.8%)	3.66	4.07	1056	3.94	3.54	1056	7.68	4.71	1056

**Table 4:** Results of multivariate and univariate analysis of variance for symptomatology

	*F*	df	*p*	*η*^2^
Sex	5.25	3	0.001***	0.010
Depression	2.02	1	0.155	0.001
Anxiety	10.19	1	0.001***	0.007
Stress	11.48	1	0.001***	0.007
Age	2.78	3	0.052	0.005
Depression	1.88	2	0.152	0.002
Anxiety	0.281	2	0.755	0.001
Stress	0.163	2	0.850	0.001
Having children	2.68	3	0.046*	0.005
Depression	5.41	1	0.020*	0.003
Anxiety	0.806	1	0.369	0.001
Stress	0.156	1	0.693	0.001
Chronically ill	6.62	3	0.001***	0.012
Depression	5.61	1	0.018*	0.003
Anxiety	18.71	1	0.001***	0.011
Stress	11.93	1	0.001***	0.007
Living with a chronically ill person	3.97	3	0.08**	0.007
Depression	9.66	1	0.002**	0.006
Anxiety	9.01	1	0.003**	0.005
Stress	9.27	1	0.002**	0.006
Family member with COVID-19	0.810	3	0.488	0.002
Depression	0.152	1	0.697	0.001
Anxiety	1.70	1	0.193	0.001
Stress	1.23	1	0.257	0.001

*p < 0.05; **p < 0.01; ***p < 0.001.

The results indicate that female teachers have higher levels of anxiety (*F*_[3,1612]_ = 10.19, *p* = 0.01, *η*^2^ = 0.007), and higher levels of stress (*F*_[3,1612]_ = 11.48, *p* = 0.01, *η*^2^ = 0.007) than their male counterparts. And although no significant differences were found as a function of age, the means of depression and stress were higher among teachers aged 23–30 years and those older than 37 years. In contrast, the mean for anxiety appeared to be higher among teachers aged 31–35 years.

With regards to parental status, teachers with children showed higher levels of depression (*F*_[3,1612]_ = 2.68, *p* = 0.046, *η*^2^ *=* 0.005) than those without, although the effect size is small. Furthermore, those who have a chronic disease showed higher levels of depression (*F*_[3,1612]_ = 5.61, *p* = 0.018, *η*^2^ = 0.003), anxiety (*F*_[3,1612]_ = 18.71, *p* = 0.001, *η*^2^ = 0.011) and stress (*F*_[3,1612]_ = 11.93, *p* = 0.001, *η*^2^ = 0.007) than those without such a disease, with anxiety being the dependent variable with the largest effect size.

Participants living with a chronically ill person also showed the highest levels of depression, anxiety and stress, all with small effect sizes (*F*_[3,1612]_ = 9.66, *p* = 0.002, *η*^2^ = 0.006), followed by stress (*F*_[3,1612]_ = 9.27, *p* = 0.002, *η*^2^ = 0.006) and anxiety (*F*_[1,1612]_ = 9.01, *p* = 0.003, *η*^2^ = 0.005). Finally, teachers with relatives infected with COVID-19 did not differ significantly in terms of any of the symptoms (see Table 4).

## Discussion

The current study explored the adverse psychological symptoms shown by teachers after 6 months of school closure due to COVID-19. We were able to recruit a large sample of teaching staff, given that the survey was conducted over a period of 1 week in the Basque Autonomous Community. Teachers of all levels participated, although the sample contained more women than men, given the higher predominance of females in the teaching profession (Tani, 2019). Almost half of the participants have school-aged children, 16.7% of the sample has chronic diseases and almost a fifth of the sample lives with someone with a chronic disease.

With regards to levels of stress, anxiety and depression, the current study shows that there is a high percentage of teachers who show adverse psychological symptomatology. First, 32.2% of the teachers showed a certain degree of depressive symptomatology. This symptomatology was mainly mild, with women showing more symptoms than men. Those older than 36 years have more depressive symptoms than their younger counterparts, although these differences were not significant. These figures are somewhat lower than those found in a study conducted in the same territory during the state of emergency (Ozamiz-Etxebarria *et al*., 2020*a*), but higher than those found in a study carried out with health professionals during the pandemic (Dosil Santamaría *et al*., 2020). According to studies carried out before the pandemic, the perceived lack of control of events and changes in work are associated with depressive symptomatology among teachers (Aznar *et al*., 2006). These factors – undoubtedly present when this study was conducted – along other variables of the pandemic that could influence psychological state such as social distancing, could explain these levels of depression among teachers (Hickie, 2020).

Furthermore, this study has also attempted to identify the other socio-personal variables that could affect the depressive symptoms experienced by teaching professionals. First, it appears that people with children are those who show significantly more depressive symptoms, even though their levels are also mild. A recent study conducted during the pandemic showed elevated maternal depression symptoms (Cameron *et al*., 2020) and this is a factor that should be considered, since depression rates could have implications for intergenerational mental health. Moreover, previous research has also suggested that single parents are particularly vulnerable to higher levels of depressive symptoms, since domestic duties can be more labour-intensive and challenging for these individuals during a pandemic (van der Velden *et al*., 2020).

As reported in previous studies (Applegate and Ouslander, 2020), people with chronic diseases show significantly more depression than those without such diseases. Those living with a chronically ill person also show significantly more mild to moderate depressive symptoms. Furthermore, those who have had a family member with COVID-19 also have higher depressive symptomatology. These findings are unsurprising, since caring for a sick person can lead to psychological symptoms (Mahoney *et al*., 2005; Peterson *et al*., 2020). In any case, depression is not the symptomatology that most concerns teachers, since it is somewhat lower than anxiety and stress symptomatology.

With regards to anxiety, almost half of the teachers (49.3%) show anxious symptomatology, and of these, most were moderate. This symptomatology is significantly higher in women than in men, as many studies have already shown (Gao *et al*., 2020; Huang and Zhao, 2020; Lai *et al*., 2020; Liu *et al*., 2020; Qiu *et al*., 2020). Comparing the results of the current study with that out in the same territory at the beginning of the pandemic with the general population (Ozamiz-Etxebarria *et al*., 2020*a*, *b*) and with health professionals in the middle of the first wave of the pandemic (Dosil Santamaría *et al*., 2020) in the current study there is a higher percentage of participants with anxious symptoms. Anxiety has indeed been a well-studied pathology among teachers (Ng, 2007; Al Lily, 2020; Besser *et al*., 2020), and during the pandemic it has been shown that this symptomatology is more severe. The uncertainty created by the lack of clear measures and resources (Lucas, 2020a; b) could be creating these anxiety levels, and, indeed, the educational community has criticised the lack of much-needed resources, guidelines and support (Navarro, 2020). Moreover, another factor that could explain this symptomatology could be that during the pandemic, teachers have been forced to adapt their teaching to online or blended modalities and the use of ICT has been significantly increased. Indeed, previous research has shown that the use of ICT can create symptoms of anxiety (Cuervo *et al*., 2018) and that for many teaching staff, their understanding of such technology is below the standards required to face this new challenge (Schleicher, 2020).

With regards to the remaining socio-personal variables, those with children have more anxiety than those without, even though the difference is not significant. Moreover, those who have a chronic illness and those who live with a person with a chronic illness show a significantly higher level of anxiety. Finally, those who live with a person who has been infected with COVID-19 also show greater levels of anxiety, possibly due to the fear of infecting the ill relative, along with the fear of being infected themselves (Baldi and Savastano, 2020).

The stress symptomatology reported in the current study is particularly worth noting since 50.4% of the surveyed teachers report having moderate stress symptoms. And again, women show significantly more stress symptoms than men. Similarly, as in the case of anxiety, higher levels of stress are observed in comparison with the beginning of the pandemic and those reported by health professionals in the middle of the pandemic (Dosil Santamaría *et al*., 2020; Ozamiz-Etxebarria *et al*., 2020*a*). It should be borne in mind that teachers have been accumulating stress since March 2020 due to, among other factors, an excessive workload, lack of clear guidelines and lack of staff and resources. Moreover, stress symptomatology has always been present in the teaching profession (Ng, 2007; Al Lily, 2020; Besser *et al*., 2020). This stress could also be due to work overload, along with interpersonal communication issues, insufficient training and job insecurity (Pérez, 2003), all of which have increased significantly during the pandemic. The findings of various international studies have shown that teachers tend to take a considerable amount of sick leave due to stress (Ryan *et al*., 2017; Von der Embse *et al*., 2019). Therefore, high levels of long-term stress could be highly detrimental to the stability of the teaching staff, which could, in turn, have a negative impact on the quality of teaching in schools. In addition, 2.4% of teachers show severe or extremely severe stress, which is a rather worrying result. Taking into account that in previous studies carried out in northern Spain, 18.6% of teachers reported symptoms of stress (García, 2017), it can be observed that in the current study there are more people with stress, being 50.4% those who have stress.

In relation to the rest of the variables, as expected, teachers with children, those with chronic diseases, or those with relatives with chronic diseases or COVID-19 have shown relatively higher levels of stress than teachers who do not face these circumstances. These differences are significant, particularly for those with chronic diseases and those who have a chronically ill family member, since these individuals are more vulnerable to the virus. Therefore, it is important for people with chronic diseases to manage their stress, since this can debilitate their immune system (Vitlic *et al*., 2014).

Given the significant levels of psychological symptomatology shown by teachers at the beginning of this unprecedented 2020–2021 academic year, there is a need for continued research that delves deeper into the mental health of this group. This would increase the possibility of providing appropriate resources to meet their needs and mitigate the negative effects of this pandemic on their emotional well-being. Similarly, we must be aware that this situation experienced by teachers has a direct impact on their students, since their emotional state could have a direct influence on both the quality of the education that they provide (Ozamiz-Etxebarria *et al*., 2020*c*) and on the emotional state of their students. It is therefore important that we are aware of the severity of this situation, and measures should be urgently taken to address the needs of the entire educational community. For example, it would be desirable to provide psychological support to help teachers to manage this new school year and to attend to the needs of their students. Teachers can also take measures to protect their mental health, such as taking care of their intimate interpersonal relationships, doing sport, not watching too much news or doing relaxation exercises. In this sense, it is necessary to agree on clear measures with the entire educational community based on scientific evidence regarding COVID-19.

However, work-related stress cannot be addressed by employees on their own. It requires attention from the government, which employs these teachers. On the one hand, in this exceptional situation, more teachers should be employed as they have to cope with more stressors than they did before the pandemic. The ratio of students per teacher should be reduced so that teachers can attend to both the educational needs of students and those related to COVID-19, such as hygiene measures, or adverse emotional states that the entire educational community is experiencing. In addition, institutions should provide education and training for teachers to be able to manage the stress created by this pandemic. It is necessary to provide support and management of work-related stress that they may be currently experiencing (Ilić and Nikolić, 2020). Teachers, therefore, need professional assistance to take care of their mental health (Scheuch *et al*., 2015) in these pandemic times.

This study also has several limitations. First, we recruited a non-probabilistic sample in which there may be a certain selection bias, since participation was voluntary, and thus only those who were particularly emotionally impacted could have participated. Therefore, future studies should include a more balanced probabilistic sample with respect to gender, and recruit participants from additional autonomous communities. Moreover, this study would have benefited from some open-ended questions to provide more explanatory information on why certain groups were struggling more than others. Finally, the study also does not allow for direct comparisons as it did not measure teachers' back-to-school stress, anxiety and depression in a non-pandemic school year, nor there was a control group of non-teachers during the same dates.

The chief strength of this study is that it provides evidence that professional support for teachers in the pandemic era is needed. In particular, it suggests the importance of addressing the emotional state of teachers in general, and the most vulnerable teachers in particular. The introduction of workshops that strengthen the emotional resources of teachers in educational centres could significantly enhance the emotional environment of schools. Such measures could then serve to protect the mental health of both teachers and students, which, in turn, would help to improve academic outcomes.
